# Endothelial Effect of Statin Therapy at a High Dose Versus Low Dose Associated with Ezetimibe

**DOI:** 10.5935/abc.20160048

**Published:** 2016-04

**Authors:** Maristela Magnavita Oliveira Garcia, Carolina Garcez Varela, Patricia Fontes Silva, Paulo Roberto Passos Lima, Paulo Meira Góes, Marilia Galeffi Rodrigues, Maria de Lourdes Lima Souza e Silva, Ana Marice Teixeira Ladeia, Armênio Costa Guimarães, Luis Claudio Lemos Correia

**Affiliations:** Escola Bahiana de Medicina e Saúde Pública (EBMSP)- FBDC, Salvador, BA - Brazil

**Keywords:** Endothelium / physiology, Cholesterol, Hydroxymethylglutaryl-CoA Reductase Inhibitors / therapeutic use, Ezetimibe, Anticholesterolemic Agents

## Abstract

**Background:**

The effect of statins on the endothelial function in humans remains under discussion. Particularly, it is still unclear if the improvement in endothelial function is due to a reduction in LDL-cholesterol or to an arterial pleiotropic effect.

**Objective:**

To test the hypothesis that modulation of the endothelial function promoted by statins is primarily mediated by the degree of reduction in LDL-cholesterol, independent of the dose of statin administered.

**Methods:**

Randomized clinical trial with two groups of lipid-lowering treatment (16 patients/each) and one placebo group (14 patients). The two active groups were designed to promote a similar degree of reduction in LDL-cholesterol: the first used statin at a high dose (80 mg, simvastatin 80 group) and the second used statin at a low dose (10 mg) associated with ezetimibe (10 mg, simvastatin 10/ezetimibe group) to optimize the hypolipidemic effect. The endothelial function was assessed by flow-mediated vasodilation (FMV) before and 8 weeks after treatment.

**Results:**

The decrease in LDL-cholesterol was similar between the groups simvastatin 80 and simvastatin 10/ezetimibe (27% ± 31% and 30% ± 29%, respectively, p = 0.75). The simvastatin 80 group presented an increase in FMV from 8.4% ± 4.3% at baseline to 11% ± 4.2% after 8 weeks (p = 0.02). Similarly, the group simvastatin 10/ezetimibe showed improvement in FMV from 7.3% ± 3.9% to 12% ± 4.4% (p = 0.001). The placebo group showed no variation in LDL-cholesterol level or endothelial function.

**Conclusion:**

The improvement in endothelial function with statin seems to depend more on a reduction in LDL-cholesterol levels, independent of the dose of statin administered, than on pleiotropic mechanisms.

## Introduction

The cardiovascular benefits of cholesterol-reducing statin therapy have been demonstrated in primary^1^ and secondary^2^ prevention scenarios, and the improvement in endothelial function is one of the involved mechanisms. This mechanism is credited to the lipid-lowering effect of the statins, supported by the association between the magnitude of the reduction in cholesterol and a reduction in cardiovascular risk.^3^ On the other hand, some authors suggest that the improvement in endothelial function is also mediated by pleiotropic actions^4,5^ independent of cholesterol: anti-inflammatory, antioxidant, and antithrombotic effects.^6-8^

These observations are based on *in vitro* studies. However, clinical confirmation has been limited by the challenge of isolating the theoretical pleiotropic effect of the statins from their lipid-lowering effect. The emergence of ezetimibe as a drug to treat hypercholesterolemia offers a scientific model suitable to test the pleiotropic hypothesis, since it allows a similar degree of reduction in LDL-cholesterol with a lower statin dose.^9^ For a similar reduction in cholesterol, higher doses of statin promoting greater endothelial benefit than smaller doses would represent clinical evidence in favor of a pleiotropic action. This model is based on the fact that ezetimibe does not interfere in the mevalonate pathway, and its effect is only mediated by the intestinal absorption of cholesterol.^10^

We conducted this randomized clinical trial to test the hypothesis that the factor influencing the endothelial function is the decrease in LDL-cholesterol, regardless of the dose of statin administered. In this study, the outcome of the statin effect on the endothelial function was evaluated by comparing the degree of arterial flow-mediated vasodilation (FMV) in individuals randomized to a high dose of simvastatin *versus* a low dose of simvastatin associated with ezetimibe.

## Methods

### Study Design

Clinical randomized, double-blind, placebo-controlled trial, registered at ClinicalTrials.gov with the identifier NCT01241097, carried out at the Obesity Outpatient Clinic of *Escola Bahiana de Medicina e Saúde Pública* in Salvador, Bahia, Brazil. The study was approved by the Ethics Committee of the institution under the protocol number 157/2009, and all participants signed a free and informed consent form.

### Cohort Selection

Women attending the clinic were consecutively selected based on the following inclusion criteria: age above 18 years, body mass index (BMI) > 25 kg/m², and LDL-cholesterol > 100 mg/dL. We defined the following as exclusion criteria: use of statin, ezetimibe, fibrate, or hormone replacement therapy within the previous 3 months; triglyceride level > 400 mg/dL; serum creatinine above 2.0 mg/dL; hepatic enzymes levels at least 1.5 times above the normal reference limit; serum creatine kinase (CPK) level higher than three times the upper normal limit; pregnancy or lactation; and occurrence of cardiac insufficiency, collagenosis, acute inflammatory conditions, or psychiatric disease. We also excluded patients who had started beta-blockers, angiotensin-conversion inhibitors, or calcium-channel blockers within the prior 4 weeks and those with a brachial artery diameter below 2.5 mm, since the measurement of the degree of dilation is compromised in these cases.

### Study Protocol

**Figure 1:**
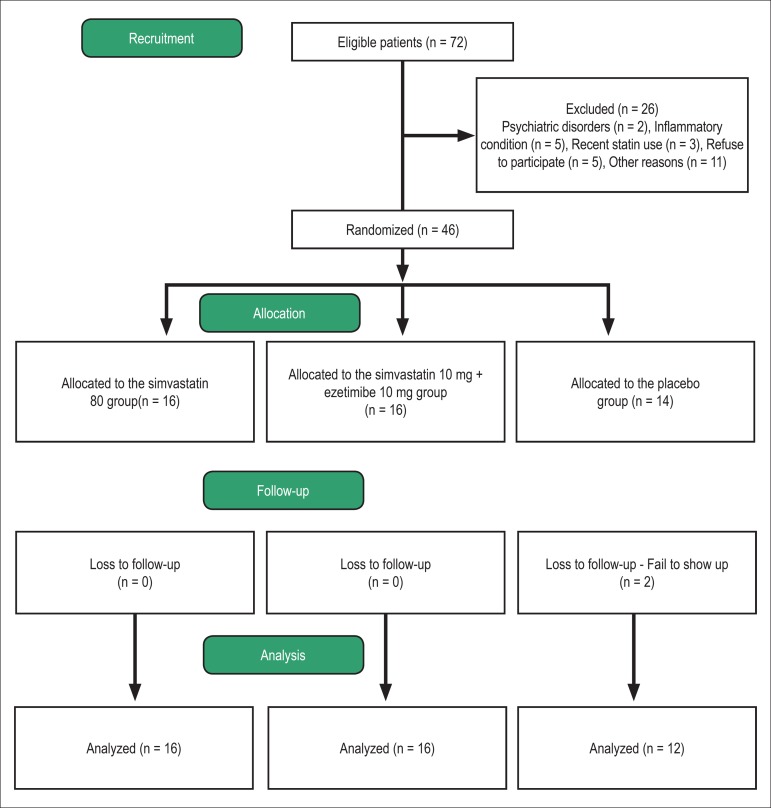
Flowchart of the study protocol.

After enrollment, the participants were randomized in blocks of three to the following treatment modalities: 1) simvastatin 80 mg, 2) simvastatin 10 mg plus ezetimibe 10 mg, and 3) placebo (Figure 1). We used the following criteria for early therapy interruption: medication intolerance, increase in liver enzymes levels three times above the upper normal level, or isolated measurement of CPK exceeding 10 times the upper normal level.

We performed three sequential evaluations to analyze the endothelial function and collect laboratory data: the first was before the beginning of the treatment, the second was after 4 weeks of treatment, and the third was after 8 weeks of treatment and represented the final assessment. During these evaluations, we recorded possible adverse events, which we rated as major (rhabdomyolysis, liver failure, renal failure, pancreatitis, obstructive jaundice, and death), intermediate (myalgia, diarrhea, and vomiting, among others), and minor (constipation, nausea and flatulence, among others).

### Biochemical Analysis

We collected blood after 12 hours of fasting following the techniques and methods standardized by the *Sociedade Brasileira de Patologia Clínica* (Brazilian Society of Clinical Pathology). We determined C-reactive protein levels with a commercially available high-sensitivity nephelometric method^11^ (Dade Behring Inc., Newark, DE, USA). Plasma concentrations of total cholesterol, HDL-cholesterol, and triglycerides were determined with a biochemical enzyme method (Dade Behring Inc., Newark, DE, USA).

### Brachial Artery Flow-Mediated Vasodilation

All participants were previously instructed to fast, not perform physical activity, drink coffee, use medications, or smoke on the day of the test. The adherence to these instructions was checked before the procedure.

We used ultrasonography with high-resolution color Doppler (Vivid 3, GE). The evaluation was performed according to a previously published guideline,^12^ and the volunteers were evaluated after fasting for 4 hours and resting while lying down for 10 minutes in a room with controlled temperature (22° to 24° C). The tests were performed by a single examiner who was blinded to the participants' data. Simultaneous electrocardiographic monitoring, coupled to the ultrasound system, allowed synchronization with the cardiac cycle. The brachial artery was identified in the longitudinal axis at 3 centimeters above the antecubital fossa and demarcated in the skin with a brush to prevent its position to change or tilt. A longitudinal image of 6 to 8 centimeters was obtained as a baseline reference. We then assessed the flow and estimated the average speed of a sample volume in the center of the artery, with a 60° vessel angulation. After that, we positioned the cuff of a sphygmomanometer in the forearm and inflated the cuff to at least 50 mmHg above the baseline systolic pressure during 5 minutes to occlude the artery. We then deflated the cuff, inducing a brief status of increased flow or reactive hyperemia, and 1 minute later obtained the image of the FMV, which represents the endothelium-dependent dilatation that occurs due to nitric oxide production caused by shear stress. We digitalized images during movement, starting 30 seconds before cuff deflation until 2 minutes later. An image corresponding to the second rest phase was acquired 15 minutes later. We then obtained again the Doppler flow of the brachial artery after releasing the cuff and 15 seconds before deflation, registering the flow speed during hyperemia. Finally, we measured the endothelium-independent vasodilation by calculating the vasodilation response 4 minutes after administration of sublingual isosorbide dinitrate 5 mg.

We digitalized all steps of the FMV assessments to analyze later the correlations between the arterial diameter at baseline and the maximum arterial diameters after dilation, as well as the FMV percentages. This analysis was conducted with 22% of the cohort, and the intraobserver correlations for these measurements were 0.99, 0.98, and 0.88, respectively, whereas the interobserver correlations were 0.98, 0.91, and 0.82, respectively. We did not perform evaluations at different moments, *i.e.* new FMV acquisitions specifically for this type of analysis.

### Data Analysis

The sample size was estimated *a priori* to achieve a statistical power of 90% ( = 5%) to detect an absolute difference of 20% in FMV variation between the treatment groups (simvastatin 80 and simvastatin 10/ezetimibe; intergroup comparison). We used the pessimistic premise that the standard deviation of the delta in each group would be around 15%, resulting in the requirement of 13 patients in each group.

The FMV was calculated as the percentage variation in artery diameter after hyperemia. The effect of the treatment on the endothelial function was measured primarily by the percentage change in FMV between baseline and after 8 weeks of treatment. This variable was compared between the two treatment groups with the Mann-Whitney test. In the intragroup analysis, FMV measurements were compared separately in each group before and after treatment with the Wilcoxon signed-rank test. For paired comparison of FMV at all three moments (baseline, 4 weeks, and 8 weeks) we used ANOVA for repeated measures. This analysis was also used to compare the treatment effects considering all three moments through an interaction between group and moment. In addition, to assess during follow-up the occurrence of possible clinical differences between the groups that could constitute confusion biases, we performed ANOVA for comparison of the clinical characteristics among the three groups.

Secondarily, we compared using the Mann-Whitney test the percentage variation in FMV between baseline and the 8th week in the active treatment groups with those in the placebo group. In this case, we opted for not comparing simultaneously the three groups (ANOVA), since this was considered a complementary analysis that did not concern the main hypothesis of the study. For group comparison at the intermediate analysis (4th week), the treatment was carried out in a similar way, since this was a complementary analysis.

We tested the linear association between the changes in LDL-cholesterol and FMV results with Spearman's correlation coefficient. We used analysis of covariance (ANCOVA) to adjust the treatment effect for age. We considered two-tailed probability values < 0.05 as statistically significant. The results are presented as mean ± standard deviation for continuous variables and as percentage for categorical variables. Variables not following a normal distribution are expressed as median and interquartile range (IQR). For statistical analyses, we used the software Statistical Package for Social Sciences, version 20 for Windows (SPSS Inc, Chicago, IL, USA).

## Results

### Characteristics of the Cohort

The cohort was characterized by young adult women (43 ± 10 years) with excess weight, evidenced by a BMI of 35 ± 5.8 kg/m^2^. Mean plasma LDL-cholesterol levels were slightly elevated (137 ± 31 mg/dL), while the median C-reactive protein level (3.6 mg/L, IQR = 1.7 - 6.7 mg/L) indicated an exacerbated inflammatory status. As for the endothelial function, the mean FMV was 8.5% ± 4.3%, including reduced and normal FMV values (healthy patients are considered to have an FMV above 7%).^13^ A diagnosis of diabetes was present in 8.7% of the participants, who were all taking metformin. Also, 41% of the participants had hypertension and were on antihypertensive drugs. None of the participants had hepatic or renal dysfunction.

Following randomization, 16 women were allocated to the simvastatin 80 group, 16 to the simvastatin 10/ezetimibe group, and 14 to the placebo group. There were no significant differences among the treatment groups regarding clinical and laboratory characteristics or class of antihypertensive drugs (Table 1). During follow-up, the clinical characteristics remained similar among the groups (Table 2). The mean FMV results were similar between the groups simvastatin 80 (8.4% ± 4.3%), simvastatin 10/ezetimibe (7.6% ± 3.9%), and placebo (9.8% ± 4.5%, p = 0.31).

**Table 1:** Comparison of clinical and laboratory characteristics among the treatment groups

	Simvastatin 80	Simvastatin 10/Ezetimibe	Placebo	p
Sample	16	16	14	
Age (years)	41 ± 8.6	48 ± 8.1	40 ± 12	0.05
BMI (kg/m^2^)	35 ± 4.3	36 ± 4.4	36 ± 8.6	0.90
Waist circumference (cm)	107 ± 7.6	108 ± 9.9	107 ±17	0.94
Waist/hip	0.92 ± 0.71	0.92 ± 0.67	0.91 ± 0.56	0.84
SBP (mmHg)	133 ± 15	132 ± 18	130 ± 18	0.86
DBP (mmHg)	85 ± 9	86 ± 13	81 ± 14	0.52
Total cholesterol (mg/dL)	205 ± 29	225 ± 47	206 ± 33	0.26
HDL-cholesterol (mg/dL)	49 ± 11	52 ± 12	49 ± 11	0.75
LDL-cholesterol (mg/dL)	133 ± 26	149 ± 43	136 ± 27	0.50
Triglycerides (mg/dL)	125 ± 51	121 ± 67	115 ± 41	0.46
CRP (mg/dL)	3.9 (2.1 – 8.1)	3.0 (1.8 – 5.1)	3.3 (1.2 – 7.2)	0.70
Blood glucose (mg/dL)	96 ± 12	103 ± 24	94 ± 19	0.39
Urea (mg/dL)	29 ± 7.3	29 ± 8.3	26 ± 6	0.42
Creatinine (mg/dL)	0.83 ± 0.11	0.79 ± 0.12	0.85 ± 0.18	0.55
AST (U/L)	18 ± 5.1	20 ± 4.6	20 ± 11	0.71
ALT (U/L)	18 ± 5.1	20 ± 4.6	20 ± 11	0.71
GGT (U/L)	32 ± 11.74	42 ± 22.99	42 ± 23.70	0.27
CPK (mg/dL)	123 ± 60	168 ± 88	109 ± 62	0.07
Hypertension	5 (31%)	6 (38%)	8 (57%)	0.33
ACEi	2 (13%)	0 (0%)	4 (29%)	0.07
ARB	1 (6.3%)	2 (13%)	2 (14%)	0.75
Menopause	1 (6.3%)	4 (25%)	2 (14%)	0.33
Smoking	0 (6.3%)	1 (6.3%)	0 (0%)	0.38
Sedentary lifestyle	11 (69%)	8 (50%)	8 (57%)	0.55
Low-calorie diet	6 (38%)	7 (44%)	6 (43%)	0.93
Diabetes	0 (0%)	1 (6.3%)	3 (21%)	0.10
Coffee consumption	14 (88%)	14 (88%)	12 (86%)	0.99
FMV	8.4% ± 4.3%	7.6% ± 3.9%	9.8% ± 4.5%	0.31

BMI: body mass index; SBP: systolic blood pressure; DBP: diastolic blood pressure; HDL: High-density lipoprotein; LDL: low-density lipoprotein; CRP: C-reactive protein; AST: aspartate aminotransferase; ALT: alanine aminotransferase; GGT: Gamma glutamyltransferase; CPK: creatine phosphokinase; ACEi: angiotensin-converting enzyme inhibitor; ARB: angiotensin receptor blocker; FMV: flow-mediated vasodilation.

**Table 2:** Comparison of the clinical characteristics in the three groups during follow-up

Characteristics	Follow-up (weeks)	Treatment groups			
		Simvastatin 80	Simvastatin 10/Ezetimibe	Placebo	p
BMI (kg/m^2^)	4 8	35 ± 4.3 34 ± 4.4	35 ± 4.4 35 ± 4.3	35 ± 6.6 36 ± 9.3	0.87 0.77
SBP (mmHg)	4 8	133 ± 13.5 133 ± 14.5	132 ± 15.3 133 ± 15.5	130 ± 15.71 32 ± 15.1	0.84 0.82
DBP (mmHg)	48	86 ± 7.3 84 ± 9.4	83 ± 9.3 84 ± 9.4	81 ± 12 81 ± 12	0.39 0.49
HDL-cholesterol (mg/dL)	4 8	49 ± 9.8 51 ± 12	53 ± 14 52 ± 13	52 ± 14 50 ± 8	0.76 0.89
Triglycerides (mg/dL)	4 8	91 ± 29 99 ± 39	124 ± 60 122 ± 73	132 ± 38 127 ± 52	0.30 0.34
Blood glucose (mg/dL)	4 8	93 ± 11 95 ± 10	102 ± 22 102 ± 18	111 ± 54 103 ± 32	0.28 0.32
Urea (mg/dL)	4 8	31 ± 5.0 29 ± 6.3	28 ± 4.7 28 ± 6.4	28 ± 4.7 27 ± 5.3	0.30 0.43
Creatinine (mg/dL)	4 8	0.84 ± 0.14 0.82 ± 0.12	0.85 ± 0.15 0.80 ± 0.12	0.78 ± 0.12 0.87 ± 0.19	0.42 0.37
AST (U/L)	4 8	17 ± 4 19 ± 5	22 ± 9 23 ± 10	17 ± 7 18 ± 5	0.17 0.17
ALT (U/L)	4 8	17 ± 6 21 ± 8	26 ± 15 25 ± 12	17 ± 10 18 ± 10	0.12 0.17
GGT (U/L)	4 8	31 ± 9.3 32 ± 11.1	41 ± 18.8 38 ± 15.3	44 ± 30.8 42 ± 33.1	0.32 0.53
CPK (mg/dL)	4 8	136 ±64 155 ± 84	185 ± 127 195 ± 118	103 ± 60 120 ± 67	0.12 0.10
CRP (mg/dL)	4 8	3.3 (2.1 – 6.4) 2.9 (1.9 – 8.3)	2.0 (1.7 – 4.2) 2.0 (1.6 – 4.2)	4.0 (2.4 – 8.2) 4.4 (2.7 – 8.3)	0.19 0.43

BMI: body mass index; SBP: systolic blood pressure; DBP: diastolic blood pressure; HDL: high-density lipoprotein; AST: aspartate aminotransferase; ALT: alanine aminotransferase; GGT: gamma glutamyltransferase; CPK: Creatine phosphokinase; CRP: C-reactive protein.

### Antilipidemic Effect of the Treatments

During the 8 weeks of the study, there were no treatment interruptions, and the adherence was complete and identical in all three groups. No side effects requiring treatment suspension were recorded. There were minor symptoms, of which the most frequent was headache (one case in the simvastatin 80 group, one case in the simvastatin 10/ezetimibe group, and three cases in the placebo group), followed by leg pain (one case in the simvastatin 80 group and one case in the simvastatin 10/ezetimibe group), and vomiting (one case in the simvastatin 10/ezetimibe group).

**Figure 2:**
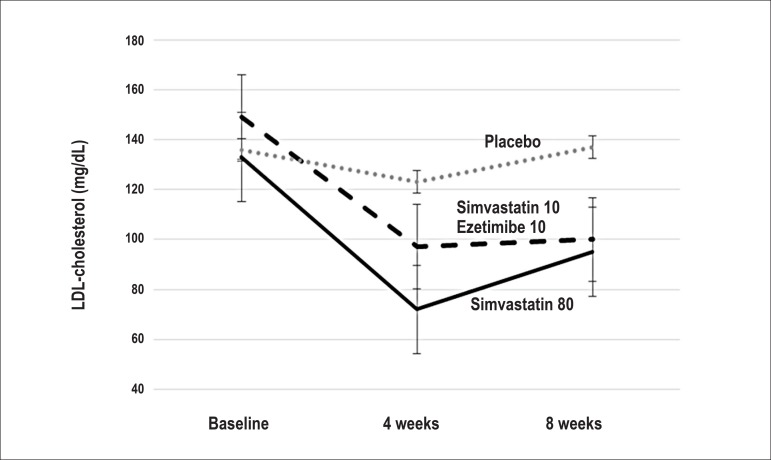
Effect of the treatments on LDL-cholesterol, showing a significant reduction of this lipoprotein in the active groups.

After 8 weeks of active treatment, there was a significant reduction in LDL-cholesterol levels, which was similar between the groups simvastatin 80 (27% ± 31%) and simvastatin 10/ezetimibe (30% ± 29%, p = 0.75). The absolute reduction was 36 ± 45 mg/dL in the simvastatin 80 group and 45 ± 36 mg/dL in the simvastatin 10/ezetimibe group (p = 0.57). There was no reduction in LDL-cholesterol levels in the placebo group (Table 3 and Figure 2).

**Table 3:** Effect of the treatments on lipid and metabolic profiles in the three groups at 4 and 8 weeks

	Baseline	8 Weeks	p	4 Weeks	p (4 *versus*8 weeks)
**Simvastatin 80**					
LDL-cholesterol (mg/dL)	133 ± 26	95 ± 44	0.006	72 ± 23	0.15
Total cholesterol (mg/dL)	205 ± 29	166 ± 48	0.007	141 ± 26	0.12
HDL-cholesterol (mg/dL)	49 ± 11	51 ± 12	0.48	49 ± 9.8	0.97
Triglycerides (mg/dL)	125 ± 51	99 ± 39	0.01	91 ± 29	0.26
Blood glucose (mg/dL)	96 ± 12	95 ± 10	0.88	93 ± 11	0.28
**Simvastatin 10/Ezetimibe**					
LDL-cholesterol (mg/dL)	149 ± 43	100 ± 45	< 0.001	97 ± 49	0.90
Total cholesterol (mg/dL)	226 ± 51	176 ± 54	< 0.001	169 ± 52	0.83
HDL-cholesterol (mg/dL)	54 ± 12	52 ± 13	0.98	53 ± 14	0.39
Triglycerides (mg/dL)	121 ± 67	122 ± 73	0.08	124 ± 60	0.52
Blood glucose (mg/dL)	103 ± 24	102 ± 18	0.28	102 ± 22	0.65
**Placebo**					
LDL-cholesterol (mg/dL)	136 ± 27	137 ± 29	0.80	123 ± 30	0.21
Total cholesterol (mg/dL)	206 ± 33	212 ± 31	0.79	201 ± 35	0.32
HDL-cholesterol (mg/dL)	49 ± 11	50 ± 8	0.39	52 ± 14	0.50
Triglycerides (mg/dL)	115 ± 41	127 ± 52	0.27	132 ± 38	0.48
Blood glucose (mg/dL)	94 ± 19	103 ± 32	0.06	111 ± 54	0.40

LDL: low-density lipoprotein; HDL: high-density lipoprotein.

The reduction in LDL-cholesterol level was already present in the assessment performed at 4 weeks of treatment, which did not differ from that performed at the 8th week in the simvastatin 80 group (p = 0.15) or in the simvastatin 10/ezetimibe group (p = 0.90).

There was no significant variation in plasma levels of HDL-cholesterol or triglycerides in any of the three treatment groups, except for a reduction in triglyceride levels in the simvastatin 80 group. Similarly, blood glucose levels remained unchanged. Liver enzymes, C-reactive protein, CPK, and weight did not change significantly during treatment. Exceptions to that were increases in CPK level in the simvastatin 80 group, which occurred without clinical complaints or values considered of risk, and ALT in the simvastatin 10/ezetimibe group (Table 4).

**Table 4:** Effect of the treatments on weight and biochemical characteristics

	Baseline	4 Weeks	p	8 Weeks	p
**Simvastatin 80**					
Body mass index (kg/m^2^)	35 ± 4.3	35 ± 4.3	0.51	34 ± 4.4	0.19
Aspartate aminotransferase (U/L)	18 ± 5.1	17 ± 4	0.73	19 ± 5	0.50
Alanine aminotransferase (U/L)	18 ± 5.1	17 ± 6	0.90	21 ± 8	0.05
Creatine phosphokinase (mg/dL)	123 ± 60	136 ±64	0.04	155 ± 84	0.03
CRP (mg/dL)	3.9 (2.1 - 8.1)	3.3 (2.1 - 6.4)	0.14	2.9 (1.9 - 8.3)	0.65
**Simvastatin 10/Ezetimibe**					
Body mass index (kg/m^2^)	36 ± 4.4	35 ± 4.4	0.63	35 ± 4.3	0.27
Aspartate aminotransferase (U/L)	20 ± 4.6	22 ± 9	0.42	23 ± 10	0.22
Alanine aminotransferase (U/L)	20 ± 4.6	26 ± 15	0.21	25 ± 12	0.03
Creatine phosphokinase (mg/dL)	168 ± 88	185 ± 127	0.30	195 ± 118	0.11
CRP (mg/dL)	3.0 (1.8 – 5.1)	2.0 (1.7 – 4.2)	0.55	2.0 (1.6 – 4.2)	0.66
**Placebo**					
Body mass index (kg/m^2^)	36 ± 8.6	35 ± 6.6	0.23	36 ± 9.4	0.81
Aspartate aminotransferase (U/L)	20 ± 11	17 ± 7	0.59	18 ± 5	0.24
Alanine aminotransferase (U/L)	20 ± 11	17 ± 10	0.20	18 ± 10	0.12
Creatine phosphokinase (mg/dL)	109 ± 62	103 ± 60	0.17	120 ± 67	0.25
CRP (mg/dL)	3.3 (1.2 – 7.2)	4.0 (2.4 – 8.2)	0.43	4.4 (2.7 – 8.3)	0.11

CRP: C-reactive protein (median and interquartile range).

### Effect of the Treatments on Arterial Flow-Mediated Vasodilation

**Figure 3:**
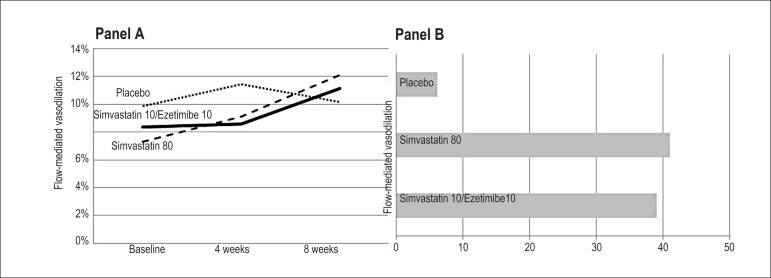
Variation in flow-mediated vasodilation, indicating an increase in the two active groups. Panel A: Line graph showing the variation in vasodilation at 4 and 8 weeks of treatment; Panel B: Bar graph showing the percentage of variation in vasodilation from baseline to the 8th week.

The simvastatin 80 group presented an increase in FMV from 8.4% ± 4.3% to 11% ± 4.2% after 8 weeks of treatment (p = 0.02). Similarly, the simvastatin 10/ezetimibe group showed improvement in vasodilation, from 7.3% ± 3.9% to 12% ± 4.4% (p = 0.001). In relative terms, the variation in arterial vasodilation had a median of +39% (IQR = 2.2% to 105%) in the simvastatin 80 group, which was similar to +41% (IQR = 13% to 227%) in the simvastatin 10/ezetimibe group (p = 0.36). This comparison remained nonsignificant after adjustment for the difference in age between these two groups (ANCOVA, p = 0.30). The placebo group presented a minimal variation in arterial vasodilation, with a median of +6.2% (IQR = - 6.6% to 56%), without statistical significance in the comparison between the baseline measurement and that performed at the 8th week (p = 0.28; Figure 3 and Table 5). When we performed a paired comparison of the three moments of evaluation (baseline, 4 weeks, and 8 weeks) with ANOVA for repeated measures, the simvastatin 80 (p = 0.045) and simvastatin 10/ezetimibe (p = 0.001) groups showed significant variations, which was different from the placebo group (p = 0.25). In this analysis, there was no interaction between group and moment when only the active treatments were considered (p = 0.30), indicating a similar variation between these two groups.

**Table 5:** Effect of the three treatments on flow-mediated vasodilation

	Baseline	8 Weeks	p	4 Weeks	p
**Simvastatin 80**					
FMV (%)	8.4 ± 4.3	11 ± 4.2	0.02	8.6 ± 3.6	0.03
FMV variation		+ 39% (2.2% – 105%)		+ 27% (-13% – 63%)	0.09
**Simvastatin 10/Ezetimibe**					
FMV (%)	7.3 ± 3.9	12 ± 4.4	0.001	9.1 ± 4.3	0.03
FMV variation		+ 41% (13% – 227%)		+ 25% (-4% – 92%)	
**Placebo**					
FMV (%)	9.8 ± 4.5	10 ± 4	0.28	11 ± 2.2	0.93
FMV variation		+ 9% (-6.6% – 56%)		+13% (-8% – 31%)	

Parentheses - 95% confidence interval. FMV: brachial artery flow-mediated vasodilation.

The active groups showed no differences in the variation in endothelium-independent vasodilation mediated by nitrate.

Unlike the effect on LDL-cholesterol, 4 weeks of treatment were not sufficient to obtain an impact on the arterial FMV comparable to that obtained at the end of 8 weeks, although a trend of improvement in vasodilation was already observed in this interim assessment. This improvement was represented by a median of +27% (IQR = - 13% to 63%, p = 0.09) in the simvastatin 80 group and +25% (IQR = - 4% to 92%, p = 0.03) in the simvastatin 10/ezetimibe group.

There was a correlation (r = - 0.33, p = 0.03) between the variations in LDL-cholesterol and FMV in a combined analysis of the studied population.

## Discussion

The present study suggests that the improvement in endothelial function promoted by statin therapy depends primarily on the drug's hypolipidemic effect, without evidence of a pleiotropic action. The pleiotropic hypothesis was tested with different doses of simvastatin (80 mg *versus* 10 mg) under the assumption that a dose-response gradient would occur if this mechanism were present. In order to avoid the degree of reduction in LDL-cholesterol as a confounding factor, ezetimibe was associated to simvastatin in the low simvastatin dose group, providing the same lipid-lowering effect as the high-dose group. When we observed that both therapies had the same benefit on the endothelial function, we inferred that the dose-response gradient was absent.

In addition to the main result, some secondary findings deserve further discussion. First, the presence of a placebo group in which the endothelial function remained unchanged assures us that the improvement observed with the active treatment in both groups was not due to a phenomenon of regression to the mean. Second, the negative correlation between the reduction in LDL-cholesterol and the improvement in endothelial function represents additional information in favor of the lipid-lowering mechanism, even though it was a weak correlation and that this analysis, as it is well known, is mainly of exploratory nature and does not show causality. Third, we observed that the positive influence on the endothelial function occurs progressively with the length of exposure, since the late results (8 weeks) were better than the early results (4 weeks), despite the fact that the nadir in LDL-cholesterol levels was achieved at 4 weeks of treatment. Regarding the anti-inflammatory mechanism, the treatments were unable to promote a reduction in C-reactive protein levels in any of the groups, which makes it less likely to be an additional mechanism of improvement in endothelial function.

The lack of differences in clinical characteristics among the groups, both at baseline (promoted by the randomization process), and during follow-up (confirmed by intergroup comparisons at 4 and 8 weeks) assured the control of possible confounding variables that could have influenced the comparative results of the outcome variable. The methodological care adopted in this study, especially that regarding the FMV analysis, also contributed to the internal validation process.

The recent study of Westerink et al.^14^ is in line with our results. In this study, the authors demonstrated a similar impact on the endothelium of simvastatin at a high dose *versus* low dose associated with ezetimibe in subjects with metabolic syndrome, as previously reported by Settergren et al.^15^ as well in patients with diabetes or coronary disease. In contrast, Liu et al.^16^ only obtained improvement in FMV with a higher dose of simvastatin and suggested the occurrence of pleiotropic benefits of the statins based on results obtained at 4 weeks. Their option to only observe for a short period of time may have precluded the observation of effects requiring longer treatment duration.

Some limitations of this study deserve recognition. The method to measure FMV followed all the steps of the protocol recommended by the International Brachial Artery Reactivity Task Force^12^. However, the method has an inherent large variability of measurements influenced by several external factors. This variability may be a limiting factor to reproduce the FMV findings and, consequently, their interpretation. The wider circumferences of the arms of obese women could have led to technical challenges in the measurements. However, this was not an interfering factor in this study since the cuff of the sphygmomanometer was positioned in the forearm, which has a shorter circumference than the arm, leaving a larger area for identification of the brachial artery when the transducer was positioned on the arm. Although the automated technique is more robust and accurate,^17^ the manual technique used in this study is also reliable and considered feasible to diagnose and monitor the endothelial function.^18^

The study was performed with a small sample, which only consisted of women with excess weight from a single outpatient clinic. However, since this is a small study, it justifies the option for homogenizing the sample and including only women. The selection of women with excess weight had the purpose of including a group more predisposed to impaired endothelial function,^19^ favoring the possibility to observe a corrective effect of the therapy. Although the choice of this type of population is justifiable, we must recognize that it reduces the generalization of the study to the overall population. We must also remember that this study has a surrogate outcome (purely mechanistic objective) that should not be interpreted as evidence that the clinical effect of the two therapies is similar. Another limitation is related to the low statistical power and refers to the fact that to avoid detecting differences among the variables we would need a very large sample, which would make the study unfeasible. Regarding the analyzes of the deltas in the general variations (including FMV and LDL-cholesterol), we found no difference among the treatments. These findings may have been influenced by a large measurement variability, hindering the statistical analysis.

## Conclusion

In conclusion, the present randomized clinical study showed that the most probable mechanism of improvement in endothelial function obtained with statins is the decrease in LDL-cholesterol, independent of the dose of statin used. In this context, the pleiotropic effects of statins have lower relevance.
